# A cyclin-dependent kinase inhibitor (p21^WAF1/CIP1^) affects thymidine incorporation in human liver cancer cells

**DOI:** 10.1038/sj.bjc.6600099

**Published:** 2002-02-12

**Authors:** Y Gong, S Deng, M Zhang, G Wang, G Y Minuk, F Burczynski

**Affiliations:** Department of Internal Medicine, Faculty of Medicine, University of Manitoba, Winnipeg, Manitoba, Canada; Department of Biochemistry and Medical Genetics, Faculty of Medicine, University of Manitoba, Winnipeg, Manitoba, Canada; Faculty of Pharmacy, University of Manitoba, Winnipeg, Manitoba, Canada

**Keywords:** p21^WAF1/CIP1^, [^3^H]thymidine incorporation, liver cancer

## Abstract

p21^WAF1/CIP1^ is a universal cyclin-dependent kinase inhibitor. To investigate the role of p21^WAF1/CIP1^ in proliferation of human liver cancer cells, we examined the expression of p53, p21^WAF1/CIP1^, cdk2 and cdk4 expression in two human liver cancer cell lines (HepG2 and PLC/PRF/5 cells). The effects of p21^WAF1/CIP1^ on [^3^H]thymidine incorporation and cdks were also examined in these cells. HepG2 cells expressed all these proteins with lower level of cdk4. PLC/PRF/5 cells expressed the other proteins except p21^WAF1/CIP1^. Transfection of p21^WAF1/CIP1^ gene inhibited [^3^H]thymidine incorporation of both cells with different extent. Although the transfection of p21^WAF1/CIP1^ did not affect cdk2 and cdk4 expression, it did reduce cdk2 kinase activity by 20%. These results suggest that: (a) p21^WAF1/CIP1^ involved in DNA synthesis of human liver cancer cells; (b) p21^WAF1/CIP1^ could be a target gene for the treatment of human hepatocellular carcinoma.

*British Journal of Cancer* (2002) **86**, 625–629. DOI: 10.1038/sj/bjc/6600099 www.bjcancer.com

© 2002 Cancer Research UK

## 

The cyclins and the cyclin-dependent kinases (cdks) are important proteins regulating the checkpoints of cell cycle progression (Hunter and Pines, 1994). In normal cells, checkpoints in the cell cycle play an important role of guiding normal cell cycle while in cancer cells, disruption of the checkpoints is responsible to abnormal growth of cancer cells. Positive and negative regulatory mechanisms control the regulation of the checkpoints in cell cycle. A cyclin-dependent kinase inhibitor (cdki) mediates one of the negative regulations of checkpoints (Sherr and Roberts, 1995). Recently, the p21^WAF1/CIP1^ gene was cloned and mapped to the chromosome 6p21.2 region (el-Deiry *et al*, 1993; Noda *et al*, 1994). p21^WAF1/CIP1^ is considered a universal cyclin-dependent kinase inhibitor. It inhibits several cyclin-cdks complex as well as DNA synthesis by inactivating proliferation cell nuclear antigen, a subunit of DNA polymerase δ (Xiong *et al*, 1993). In addition, p21^WAF1/CIP1^ involves in induction of cell differentiation (Skapek *et al*, 1995) and inhibition of tumour cell proliferation (el-Deiry *et al*, 1994).

Mutation of p21^WAF1/CIP1^ is rare in different types of human malignancy, therefore, it is suggested that p21^WAF1/CIP1^ exerts its role in tumorigenesis mainly on expression level. It seems to be true in human hepatocellular carcinoma (HCC). Several groups studying expression of p21^WAF1/CIP1^ in human hepatocellular carcinoma have documented that there were reduced p21^WAF1/CIP1^ mRNA and protein levels in human HCC (Hui *et al*, 1997; Naka *et al*, 1998; Qin *et al*, 1998; Shi *et al*, 2000). These studies suggested that disruption of p21^WAF1/CIP1^ and cell cyclin-cdks complexes may contribute to malignant progression of HCC. However, the direct role of p21^WAF1/CIP1^ in human HCC cells has not been explored. In this study, we employ an expression vector of p21^WAF1/CIP1^ to examine the direct effect of p21^WAF1/CIP1^ on human liver cancer cells.

## MATERIALS AND METHODS

### Material

Minimum Eagle's medium (MEM), sodium bicarbonate, sodium pyruvate, penicillin-streptomycin, Trypsin-EDTA and LipofectinAMINE were purchased from GIBCO/BRL (Life Technologies, Burlington ON, Canada). Dr Alan McLachlan (Research Institute of Scripps Clinic at La Jolla, CA, USA) kindly provided PLC/PRF/5 human HCC cells. HepG2 cells were purchased from ATCC (Rockville, MD, USA). Cool calf 1 and the other chemicals were purchased from Sigma Co. (St. Louis, MO, USA). The mammalian expression vector pCEP was purchased from Invitrogen (Carlsbad, CA, USA). p21^WAF1/CIP1^ and p53 antibodies were purchased from Santa Cruz Biotechnology Inc. (Santa Cruz, CA, USA). cdk2 and cdk4 antibodies, rabbit anti-mouse IgG and protein A/agarose were purchased from Transduction Laboratories (Lexington, KY, USA).

### Cell culture

Two human liver cancer cell lines were employed because they represent different states of differentiation. HepG2 is a well-differentiated cell line and derived from human hepatoblastoma. PLC/PRF/5 is a poorly differentiated cell line and derived from HCC of a patient with HBV. HepG2 and PLC/PRF/5 cells were grown in MEM containing 5% Cool Calf 1 (Sigma Co. St. Louis, MO, USA) supplemented with 10 mmol L^−1^ L-glutamine, 1 mmol L^−1^ sodium pyruvate, 100 IU ml^−1^ penicillin and 100 μg ml^−1^ streptomycin (GIBCO–BRL, Burlington, ON, Canada) in Falcon 75 cm flasks. Cultures were maintained at 37°C in a humidified atmosphere of 95% O_2_ and 5% CO_2_.

### RNA extraction and Northern blot analyses

Total RNA of HepG2 and PLC/PRF/5 cells was extracted by a Lithium chloride/Urea method (Gong *et al*, 1995) and the polyA-RNA was isolated by employing oligo-dT cellular column (Aviv and Leder, 1972). Northern blot analysis was performed using α-^32^P-dCTP-labelled full-length human p21^WAF1/CIP1^ cDNA and β-actin probes as previously described (Gong *et al*, 1995). Briefly, 6 μg of polyA- RNA was separated through 1% agarose gel, transferred onto GT-zeta nylon membrane (Bio-Rad, Burlington, ON, Canada), hybridized overnight with the probes at 42°C and washed as per the manufacturer's instructions.

### Transient transfection

1×10^5^ cells were seeded in 6-well plate one day before the transfection. Mock (water), pCEP or pCEP-WAF1 was mixed with LipofectinAMINE and the mixtures were then incubated with cells for 24 h. After 24 h, cells were washed and incubated with culture media without antibiotics for a further 24 h followed by culturing in completed media for 36 h to allow expression of p21^WAF1/CIP1^ gene.

### Cell proliferation assay

For cell doubling time, both cell lines were plated at 1×10^5^ cells in 6-well plates, cell numbers were counted at days 2 and 6 after seeding. Cell doubling time was calculated according to the formula (doubling time=*t*×log 2/log Nt/Ni, where *t*=time between the count, Nt=cell number at day 6 and Ni=cell number at day 2) as described previously (Gong *et al*, 1991). For ^3^H-thymidine incorporation assays, cells transfected as delineated above were labelled with 10 μCi of ^3^H-thymidine (specific activity 45 Ci mmol^−1^, Amersham, Oakville, ON, USA) for 2 h, fixed in 10% trichloroacetic acid and lysed in 400 μl of 0.2M sodium hydroxide. One hundred μl of the cell lysate was employed to measure [^3^H]thymidine incorporation with a LKB liquid scintillation counter (Wallac, Turku, Finland) and 10 μl of the cell lysate was used to measure protein content by the Lowry technique.

### The Western blot analyses

Cells non-transfected or transfected with mock, pCEP vector, and pCEP-WAF1 as delineated above were lysed in 100 μl of 2× loading buffer (125 mmol L^−1^ Tris-HCl, pH 6.8, 4% SDS, 20% glycerol, 0.1% bromophenol blue and 2.5% β-mercaptoethanol). Twenty μg of cell lysate from each sample was separated in a 15% polyacrylamide/SDS gel, transferred onto Nitroplus-2000 membranes (Micron Separations Inc. Westborough, MA, USA) and incubated with antibodies against p53, p21^WAF1/CIP1^, cdk2 and cdk4 respectively. Immunoreactive bands were visualized by using the ECL (enhanced chemiluminescence) detection method (Amersham, Arlington Heights, IL, USA) (Gong *et al*, 1995).

### Immunokinase assay

Immunokinase assay was performed following a protocol from Transduction Laboratories (Lexington, KY, USA). Briefly, cells with no transfection and transfection of mock, pCEP vector, and pCEP-WAF1 as described above were lysed in 1 ml cold lysis buffer (10 mmol L^−1^ Tris-HCl pH 7.4, 1.0% Triton X-100, 0.5% Nonidet P-40, 150 mmol L^−1^ NaCl, 20 mmol L^−1^ Sodium fluoride, 0.2 mmol L^−1^ sodium ortho-vanadate, 1.0 mmol L^−1^ EDTA, 0.2 mmol L^−1^ PMSF) for 30 min at 4°C. One mg of the total cell lysate was employed to incubate with 5 μg of mouse monoclonal antibodies against cdk2 and then with the same amount of rabbit anti-mouse IgG. After centrifugation, the immune complexes were further incubated with 10 μl of the 50% protein A : agarose suspension. The immunocomplexes were suspended in 40 μl of kinase buffer (10 mmol L^−1^ Tris-HCl pH 7.4, 150 mmol L^−1^ NaCl, 10 mmol L^−1^ MgCl_2_, 0.5 mmol L^−1^ DTT) with 25 μM ATP, 2.5 μCi [^32^P-γ]-ATP and 1 mg ml^−1^ histone 1 and then incubated at 37°C for 15 min. The reactions were then electrophoresed on 15% SDS–PAGE and kinase activities were indicated by the band of histone 1.

## RESULTS

**Figure 1:**
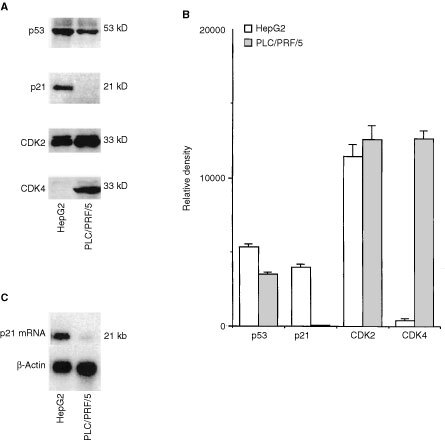
Expression of p53, p21^WAF1/CIP1^, cdk2 and cdk4 in HepG2 and PLC/PRF/5 cell lines. (**A**) Shows Western blot analysis of p53, p21^WAF1/CIP1^, cdk2 and cdk4 in HepG2 and PLC/PRF/5 cells. Twenty μg of each cell lysate was resolved in 15% polyacrylamide/SDS gel and incubated with antibodies against p53, p21^WAF1/CIP1^, cdk2 and cdk4 respectively. (**B**) Shows histogram of densitometry of Western blot analyses of p53, p21^WAF1/CIP1^, cdk2 and cdk4 from four individual experiments (mean±s.e.). (**C**) Shows Northern blot analysis of p21^WAF1/CIP1^ mRNA abundance in HepG2 and PLC/PRF/5 cells. Six μg of polyA- RNA from each cell line was separated in 1% agarose and hybridized with the full length human p21^WAF1/CIP1^ cDNA and β-actin cDNA probes respectively.

**Figure 2:**
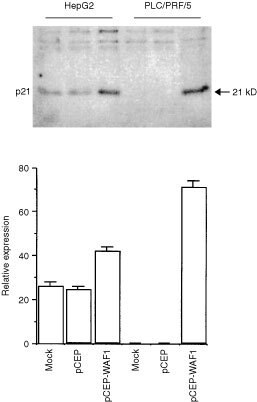
The upper panel shows Western blot analyses of transfected p21^WAF1/CIP1^ protein in HepG2 and PLC/PRF/5 cells. Both cell lines were transfected with mock (with water), pCEP (2 μg) and pCEP-WAF1 (2 μg). Twenty μg of each cell lysate was resolved in 15% SDS–PAGE and transferred onto Nitroplus-2000 membrane. The membranes were then incubated with antibody against p21^WAF1/CIP1^. The lower panel shows histogram of densitometry of Western blot analyses of transfected p21^WAF1/CIP1^ protein in both cell lines. The data represents mean±s.e. (*n*=4).

We employed two human liver cancer cell lines to investigate the direct effect of p21^WAF1/CIP1^ on these cells. The two human liver cancer cell lines – HepG2 and PLC/PRF/5 exhibited very different expression pattern of p53, p21^WAF1/CIP1^, cdk2 and cdk4. By Western blot analyses, p53, p21^WAF1/CIP1^, cdk2 and cdk4 can be detected in HepG2 cells while p53, cdk2 and cdk4 were observed in PLC/PRF/5 cells ( Figure 1A

). Moreover, the abundance of p53 protein in HepG2 cells was higher than that of PLC/PRF/5 cells. Furthermore, cdk4 protein level was higher in PLC/PRF/5 cells than in HepG2 cells (Figure 1B). Although p21^WAF1/CIP1^ protein was not observed in PLC/PRF/5 cells, its mRNA was expressed at low level in PLC/PRF/5 cells as compared to that in HepG2 cells (Figure 1C). Furthermore, the expression of p21^WAF1/CIP1^ was related to longer cell doubling time (HepG2 cells 44±4 h *vs* PLC/PRF/5 cells 29±3 h, *P*<0.05). Under the same transfection condition, the p21 level as the result of transfection in PLC/PRF/5 cells was four times than that in the HepG2 cells. However, the total p21 levels in PLC/PRF/5 cells after transfection was about twice of that in the HepG2 cells ( Figure 2

).

**Figure 3:**
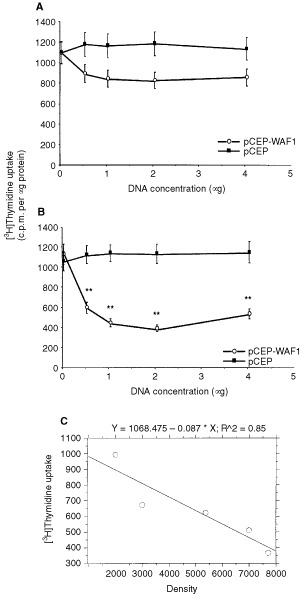
The effect of p21^WAF1/CIP1^ on [^3^H]thymidine incorporation in HepG2 and PLC/PRF/5 cells. (**A**) Shows the effect of different concentrations of pCEP and pCEP-WAF1 on [^3^H]thymidine incorporation in HepG2 cells. The data represents mean±s.e. (*n*=8). (**B**) Shows the effect of different concentrations of pCEP and pCEP-WAF1 on [^3^H]thymidine incorporation in PLC/PRF/5 cells. The data represents mean±s.e. (*n*=8). **Indicates *P*<0.01. (**C**) Displays correlation between densities of p21^WAF1/CIP1^ and [^3^H]thymidine uptake in PLC/PRF/5 cells. Cells were transfected with 0.1, 0.5, 0.75, 1 and 2 μg of p21^WAF1/CIP1^ cDNA. The densities of p21^WAF1/CIP1^ protein and [^3^H]thymidine uptake were plotted by StatView regression program. There is a statistically significant correlation between thymidine uptake and density (*P*=0.0258).

The effects of p21^WAF1/CIP1^ on cell proliferation of two human liver cancer cells were shown in Figure 3

**Figure 4:**
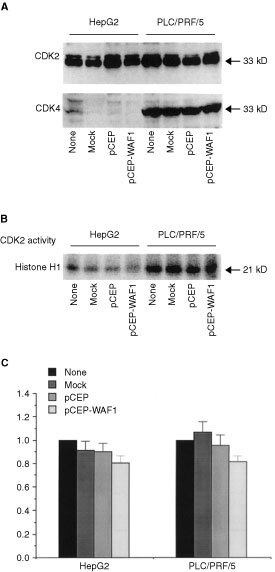
Both HepG2 and PLC/PRF/5 cells were treated as indicated – none – no transfection, mock – transfection with water, pCEP vector and pCEP-WAF1. (**A**) Shows the effect of p21^WAF1/CIP1^ on cdk2 and cdk4 protein abundance in these cells. Twenty μg of each cell lysate was used for Western blot analyses and the experiment was repeated on three occasions. (**B**) Shows the effect of cdk2 kinase activity in these cells. The cell lysates (1 μg of each) were immunoprecipitated with anti-CDK2 antibody and the immunoprecipitates were incubated with a reaction solution for *in vitro* phosphorylation of histone H1 as described in Materials and Methods. Samples were then electrophoresis and phosphorylated histone 1 was visualized by autoradiography of the slab gels. (**C**) Displays the histogram of cdk2 kinase activity. The data was generated from four separated experiments and represented as mean±s.e. The no transfection was arbitrarily set as 1.

. The p21^WAF1/CIP1^ inhibited cell proliferation of both HepG2 and PLC/PRF/5 cells with different extent. Although lower than pCEP vector control, the p21^WAF1/CIP1^ did not significantly inhibit HepG2 cell proliferation (Figure 3A). However, the p21^WAF1/CIP1^ inhibited PLC/PRF/5 cell proliferation in a dose-dependent manner. Progressive increases in the amount of transfected p21^WAF1/CIP1^ cDNA resulted in a progressive inhibition of PLC/PRF/5 cell proliferation until maximum inhibition was obtained at 2 μg of the p21^WAF1/CIP1^ cDNA. When compared to cell proliferation associated with transfection of the pCEP vector alone, the differences were statistically significant (*P*<0.01, Figure 3B). Moreover, inhibition of [^3^H]thymidine incorporation was correlated with the expression of p21^WAF1/CIP1^ protein in PLC/PRF/5 cells (Figure 3C). To elucidate the mechanism of p21^WAF1/CIP1^ inhibition of cell proliferation, we examined the p21^WAF1/CIP1^ regulation of cdk2 and cdk4 in both PLC/PRF/5 and HepG2 cells. Our results showed that the p21^WAF1/CIP1^ did not alter either cdk2 or cdk4 proteins in these cells ( Figure 4A

). The cdk2 kinase activity was further examined by immunokinase assay. Although cdk2 kinase activity was higher in PLC/PRF/5 cells than that in HepG2 cells, the p21^WAF1/CIP1^ reduce cdk2 kinase activity by 20% in both cells (Figure 4B).

## DISCUSSION

p21^WAF1/CIP1^ was identified as a potential mediator of p53 protein and a co-immunoprecipitated protein of cyclin D1 (Xiong *et al*, 1992; el-Deiry *et al*, 1993). Extensive studies reveal that p21^WAF1/CIP1^ involves in the progression of cell cycle. It inhibits several cyclin-cdk activities and also is a component of proliferating nuclear antigen (PCNA) and DNA polymerase δ complexe (Sherr and Roberts, 1995). The p21^WAF1/CIP1^ protein contains two functional domains, which are mediated in the inhibition of cdk kinase and binding of PCNA. The amino end of p21^WAF1/CIP1^ contains an inhibitory domain of cdk, which inhibits cyclin D and E associated cdks activities. The carboxyl end of p21^WAF1/CIP1^ mediates the PCNA-binding and activity of DNA polymerase δ (Chen *et al*, 1995; Luo *et al*, 1995; Nakanishi *et al*, 1995; Warbrick *et al*, 1995). Our results showed that p21^WAF1/CIP1^ inhibited [^3^H]thymidine incorporation and reduced cdk2 kinase activity in human liver cancer cells but did not affect expression of cdk2 and cdk4. The findings suggest that the transfected p21^WAF1/CIP1^ likely has no apparent effect on the expression of cdks but may block the ability of PCNA to activate DNA polymerase δ and the cdk kinase activity in human liver cancer cells. The distinguished action of p21^WAF1/CIP1^ was demonstrated in a SV40-based DNA replication system. In this *in vitro* DNA replication system, p21^WAF1/CIP1^ was able to block PCNA-activated DNA polymerase δ activity without the participation of cyclin-bound cdks (Flores-Rozas *et al*, 1994; Waga *et al*, 1994).

The role of p21^WAF1/CIP1^ in tumorgenesis of human hepatocellular carcinoma (HCC) is not clear. However, reduction of p21^WAF1/CIP1^ mRNA and protein abundance was observed in human HCC (Naka *et al*, 1998; Qin *et al*, 1998). Studies of p21^WAF1/CIP1^ mRNA abundance in human HCC from Chinese and Japanese groups showed some controversies (Naka *et al*, 1998; Qin *et al*, 1998). The difference could be due to the infection of different hepatitis viruses as well as non-viral infection. One of the Japanese groups showed that HCC developed from p53-altered and HCV infected patients exhibited reduced p21^WAF1/CIP1^ protein abundance while there was little change of p21^WAF1/CIP1^ protein level in those patients with HBV infection or no viral infection (Shi *et al*, 2000). Their finding was supported by that HCV core protein inhibited the promoter activity of p21^WAF1/CIP1^ gene (Ray *et al*, 1998). But the reduction of p21^WAF1/CIP1^ mRNA abundance in HCC could be more complicated because of p53 gene status in HCC. In this study, we employed a HCC cell line – PLC/PRF/5 cells, which were derived from an African man with HBV infection and carry HBV genome (Aden *et al*, 1979; Koch *et al*, 1984). However, we observed that there was no expression of p21^WAF1/CIP1^ protein and the induced expression of p21^WAF1/CIP1^ protein significantly inhibited [^3^H]thymidine incorporation in PLC/PRF/5 cells. Because there is a mutation of p53 gene in PLC/PRF/5 cells (Hsu *et al*, 1993), the absence of p21^WAF1/CIP1^ protein in PLC/PRF/5 cells could be related to p53 gene rather than viral infection. It is consistent with HepG2 cells because HepG2 cells carry wild type p53 gene and no HBV genome (Hosono *et al*, 1991). In these cells, the p21^WAF1/CIP1^ level is higher than that in PLC/PRF/5 cells. Therefore, the p21^WAF1/CIP1^ mRNA abundance in human HCC may be dependent on both the type of viral infection and p53 gene status.

One of our findings was that the expression of p21^WAF1/CIP1^ protein inhibited [^3^H]thymidine incorporation in human liver cancer cells no matter of whether there was viral infection or mutation of p53 genes. The difference between HepG2 and PLC/PRF/5 cells was the degree of inhibition of [^3^H]thymidine incorporation. This can be due to the ratio of p21^WAF1/CIP1^ and cdks because the ratio of p21^WAF1/CI^ and cdks could determine whether the p53 deficient cancer cells proliferated or not (Zhang *et al*, 1994; Shima *et al*, 1998). In our case, that PLC/PRF/5 cells expressed no p21^WAF1/CIP1^ but higher cdk4 level made it more susceptible to the inhibitory effect of p21^WAF1/CIP1^. HepG2 cells expressed p21^WAF1/CIP1^ and had lower cdk4 level, therefore, HepG2 cells were less sensitive to the effect of p21^WAF1/CIP1^.

In conclusion, our present study has shown that: (a) HepG2 and PLC/PRF/5 have distinguished expression pattern of p53, p21^WAF1/CIP1^, and cdk4; (b) although both cells exhibited the same level of cdk2, activity of cdk2 was higher in PLC/PRF/5 cells than in HepG2 cells; (c) p21^WAF1/CIP1^ inhibited [^3^H]thymidine incorporation and cdk2 kinase activity in both HepG2 and PLC/PRF/5 cells, however, the most significant inhibition was observed in PLC/PRF/5 cells; (d) since p21^WAF1/CIP1^ inhibited DNA synthesis of human liver cancer cells, p21^WAF1/CIP1^ could be a target gene for the treatment of human HCC.
